# Editorial: Stress and Stress Management – Pushing Back Against Existing Paradigms

**DOI:** 10.3389/fpsyg.2022.859660

**Published:** 2022-02-25

**Authors:** Matthew J. Grawitch, Larissa K. Barber, Michael P. Leiter, Joseph J. Mazzola

**Affiliations:** ^1^School for Professional Studies, Saint Louis University, St. Louis, MO, United States; ^2^Department of Psychology, San Diego State University, San Diego, CA, United States; ^3^Department of Psychology, Acadia University, Wolfville, NS, Canada; ^4^Department of Psychology, Meredith College, Raleigh, NC, United States

**Keywords:** stress, stress response, appraisals, wellbeing, editorial

When we originally set out this special issue, the goal was to identify assumptions, claims, and inferences within existing work stress paradigms that may not be evidence based even though they have become accepted within the field as valid or true. Additionally, we sought to get a sense of new or innovative ways in which researchers may be considering the domain of stress in organizations. To that end, we put out a call for articles, and the special issue ended up with five compelling articles, ranging from theoretical (Horan et al. and Pindek fall into this domain) to empirical (Cropley and Collis; Huang et al.; Sonnentag and Nieesen fall into this domain) contributions. When looking across the articles in this issue, we identified three broad contributions.

## Appraisals of Stress are Unique Rather Than Universal

There is often an assumption that assessing work environment stressors objectively or universally is a superior way to understand employee stress. When it comes to understanding employee stress, however, a psychological approach that centers appraisals in the stress process is critical. This contribution was evident in all five of the articles but really emphasized in the articles presented by Horan et al. and Pindek. Pindek's article focuses on job underperformance as a potential stressor, whereas Horan et al. provides a review of the Challenge-Hindress Stress Model (CHM), but both strongly emphasized the role of appraisals in the experience of stress. Pindek argued that while objective measures of performance exist, it is how individuals appraise their performance, whether acutely or chronically, that may result in increased stress. This is a useful contribution to occupational health research approaches that often conceptualize feedback as a positive resource driving work engagement (Bakker and Demerouti, 2017). Yet the nature of the feedback in relation to performance expectations can be a driving force behind negative motivational and emotional outcomes for employees.

Horan et al. argue that an a priori classification of challenge or hindrance stressors ignores the relevance of appraisals. That is, there is often an assumption of some work demands being almost universally motivating (time pressure, workload) given they facilitate growth or performance, whereas others can be demoralizing or distressing given they are perceived as obstacles to performance (bureaucratic red tape, interruptions). But this classification strategy for demands ignores important contextual information (e.g., person, environment) that can change those appraisals for employees. Moreover, treating these appraisals as mutually exclusive and static has also undermined our knowledge and study designs in this area. People can and do see the same demand as both a challenge and a hindrance, and these perceptions can change over time.

## When and Why Thinking About Work Helps or Hurts

Beyond understanding actual work demands or stressors in the workplace, our ongoing thoughts about work, long after we leave the workplace, are also important. Cropley and Collis and Sonnetag and Niessen provide insights into both when and why thinking about work helps or hurts. Mentally, switching off from work is often considered an overwhelmingly good thing - an important recovery process critical to our health (Sonnentag and Fritz, 2015). But this may not always be the case. Sonnetag and Niessen focus on the issue of psychological detachment from work in a sample of students and employees. Although they indeed found that detachment is a useful way to reduce post-work negative affect, detachment also resulted in lower post-work positive affect.

Additionally, it is often assumed that thinking about work is necessarily unhealthy, but thoughts about work are not always negative. Unsurprisingly, Sonnetage and Niessen found that thinking negatively about work was the worst for both positive and negative affect. However, they also found that thinking positively about the workday resulted in similar levels of reduced negative affect and higher levels of positive affect as did detachment. Thus, cutting our thoughts off from work when things are going well may not be best for us.

Cropley and Collis also challenged our assumptions of *why* employees may be experiencing negative thoughts about work (or rumination). We often assume these thoughts arise from high job demands, more fatigue, or poor sleep. However, their two studies found that a better explanation may be a decrease in executive function, specifically with regard to the ability to shift focus and attention away from work thoughts. This has important implications for developing interventions that target both increasing focus and inhibition to avoid negative thoughts.

## Leadership Matters for Work Engagement

It is often assumed that stress levels are primarily the result of work characteristics, but the article by Huang et al. suggests the social aspect of work, specifically as it relates to leadership, plays an important role as well. They find that employee perceptions of transformational, ethical, and participative leadership may be important for stimulating work engagement among employees and may help to minimize the likelihood of counterproductive work behaviors. Perceptions of leadership may have implications for both acute and chronic underperformance. As noted by Pindek, those who are more engaged may be less likely to experience recurring issues of underperformance. It is also possible that such relationships could play a role in some of the issues discussed by Horan et al., specifically in terms of the way subordinates appraise demands as either challenges or hindrances, which could then have consequences for engagement or counterproductive work behaviors.

## Concluding Thoughts

Here, we discussed what we identified as three broad contributions made by the articles in this special topic. Although at first they might seem to be unrelated to each other, the issues discussed in one article often pose implications for issues discussed in one or more of the other articles. The appraisal process has direct implications for how people make sense of their participation in the workplace. Further, employees' understanding of their workplace experiences are shaped through their relationships with leaders that signal key workplace values. Although the articles in this topic help to broaden our understanding of the stress process, there is more work to be done. Whether through the lens of the Job Demands-Resources Model (Demerouti et al., 2001; Bakker and Demerouti, 2017), Conservation of Resources theory (Hobfoll, 1989), or some other theoretical perspective, we expect future research will be able to build off of the theoretical and empirical claims put forth in this issue to better refine our understanding of the stress process.

## Author Contributions

ML and JM assisted in refining the article. All authors contributed to the article and approved the submitted version.

## Conflict of Interest

The authors declare that the research was conducted in the absence of any commercial or financial relationships that could be construed as a potential conflict of interest.

## Publisher's Note

All claims expressed in this article are solely those of the authors and do not necessarily represent those of their affiliated organizations, or those of the publisher, the editors and the reviewers. Any product that may be evaluated in this article, or claim that may be made by its manufacturer, is not guaranteed or endorsed by the publisher.
